# Piperaquine resistant Cambodian *Plasmodium falciparum* clinical isolates: in vitro genotypic and phenotypic characterization

**DOI:** 10.1186/s12936-020-03339-w

**Published:** 2020-07-25

**Authors:** Nonlawat Boonyalai, Brian A. Vesely, Chatchadaporn Thamnurak, Chantida Praditpol, Watcharintorn Fagnark, Kirakarn Kirativanich, Piyaporn Saingam, Chaiyaporn Chaisatit, Paphavee Lertsethtakarn, Panita Gosi, Worachet Kuntawunginn, Pattaraporn Vanachayangkul, Michele D. Spring, Mark M. Fukuda, Chanthap Lon, Philip L. Smith, Norman C. Waters, David L. Saunders, Mariusz Wojnarski

**Affiliations:** 1 Department of Bacterial and Parasitic Diseases, Armed Forces Research Institute of Medical Sciences, 315/6 Rajvithi Road, Bangkok, 10400 Thailand; 2 Walter Reed Army Institute of Research, Silver Spring, Maryland, 20910 USA; 3 U.S. Army Research Institute of Infectious Diseases, Frederick, MD USA

**Keywords:** Drug combination, Exonuclease, Malaria, PfCRT, Piperaquine resistance, Plasmepsin

## Abstract

**Background:**

High rates of dihydroartemisinin–piperaquine (DHA–PPQ) treatment failures have been documented for uncomplicated *Plasmodium falciparum* in Cambodia. The genetic markers plasmepsin 2 (*pfpm2*), exonuclease (*pfexo*) and chloroquine resistance transporter (*pfcrt*) genes are associated with PPQ resistance and are used for monitoring the prevalence of drug resistance and guiding malaria drug treatment policy.

**Methods:**

To examine the relative contribution of each marker to PPQ resistance, in vitro culture and the PPQ survival assay were performed on seventeen *P. falciparum* isolates from northern Cambodia, and the presence of E415G-Exo and *pfcrt* mutations (T93S, H97Y, F145I, I218F, M343L, C350R, and G353V) as well as *pfpm2* copy number polymorphisms were determined. Parasites were then cloned by limiting dilution and the cloned parasites were tested for drug susceptibility. Isobolographic analysis of several drug combinations for standard clones and newly cloned *P. falciparum* Cambodian isolates was also determined.

**Results:**

The characterization of culture-adapted isolates revealed that the presence of novel *pfcrt* mutations (T93S, H97Y, F145I, and I218F) with E415G-Exo mutation can confer PPQ-resistance, in the absence of *pfpm2* amplification. In vitro testing of PPQ resistant parasites demonstrated a bimodal dose–response, the existence of a swollen digestive vacuole phenotype, and an increased susceptibility to quinine, chloroquine, mefloquine and lumefantrine. To further characterize drug sensitivity, parental parasites were cloned in which a clonal line, 14-B5, was identified as sensitive to artemisinin and piperaquine, but resistant to chloroquine. Assessment of the clone against a panel of drug combinations revealed antagonistic activity for six different drug combinations. However, mefloquine-proguanil and atovaquone–proguanil combinations revealed synergistic antimalarial activity.

**Conclusions:**

Surveillance for PPQ resistance in regions relying on DHA–PPQ as the first-line treatment is dependent on the monitoring of molecular markers of drug resistance. *P. falciparum* harbouring novel *pfcrt* mutations with E415G-exo mutations displayed PPQ resistant phenotype. The presence of *pfpm2* amplification was not required to render parasites PPQ resistant suggesting that the increase in *pfpm2* copy number alone is not the sole modulator of PPQ resistance. Genetic background of circulating field isolates appear to play a role in drug susceptibility and biological responses induced by drug combinations. The use of latest field isolates may be necessary for assessment of relevant drug combinations against *P. falciparum* strains and when down-selecting novel drug candidates.

## Background

In 2018, an estimated 228 million cases of malaria resulted in approximately 405,000 deaths globally [1]. Artemisinin-based combination therapy (ACT), composed of potent, short-acting artemisinin derivatives and longer-acting partner drugs, provide the first-line drug treatment for uncomplicated *Plasmodium falciparum* infection for most malaria endemic areas. However, the spread of drug resistance will require that current anti-malarial drugs be replaced with newer chemotherapeutic agents [2] or more effective drug combinations. Within a few years of dihydroartemisinin-piperaquine (DHA–PPQ) being recommended by the World Health Organization (WHO) as first-line therapy for uncomplicated *P. falciparum* malaria [3], the emergence of DHA–PPQ resistance was reported in Cambodia [4–7], Vietnam [8] and more recently in Thailand [9], where DHA–PPQ is still the first-line ACT for *P. falciparum* treatment in most provinces.

Artemisinin (ART) resistance is characterized by delayed parasite clearance times [10, 11] and is associated with mutations in the propeller domain of *P. falciparum* Kelch-13 gene (*pfkelch13*) [12–15]. ART resistance can also be confirmed by the Ring-stage Survival Assays (RSA) (in vitro RSA^0−3h^ and ex vivo RSA) [12, 16]. The characterization of PPQ resistance with conventional ex vivo*/*in vitro dose response assay has been challenging, yielding wide IC_50_ values [5, 17]. The PPQ survival assay (PSA) has been developed, with a survival rate of more than 10% defining a PPQ resistant phenotype [18]. Genetic markers proposed as modulators of PPQ resistance include *P. falciparum* multidrug resistance 1 (*pfmdr1*) (PF3D7_0523000) [19–21], *P. falciparum* plasmepsin 2 (*pfpm2*) (PF3D7_1408000) [20–23], *P. falciparum* exonuclease (*pfexo*) (PF3D7_1362500) [20, 23, 24], and specific mutations on *P. falciparum* chloroquine resistance transporter (*pfcrt*) (PF3D7_0709000) [18, 25–27]. An association between in vitro PPQ-resistant isolates and single-copy *pfmdr1* was found [6, 7, 18, 28]; however, not all single copy *pfmdr1* isolates demonstrate in vitro PPQ resistance. Ansbro et al. [29] have shown that *pfmdr1* amplifications were absent in all Cambodian *P. falciparum* samples from 2014 to 2015, which could reflect limited use of mefloquine during that period [20]. Two independent genome-wide association studies (GWAS) showed that amplification of the *pfpm2* gene was associated with reduced PPQ sensitivity [20, 21]. Targeted gene disruption of either *pfpm2* or *pfpm3* in *P. falciparum* 3D7 background accounted for a slight decrease in PPQ IC_50_ values, and a significant increase in sensitivity to PPQ in a modified PSA [30]. However, the overexpression of *pfpm2* and *pfpm3* in the 3D7 genetic background did not alter the sensitivity of *P. falciparum* to PPQ, suggesting that the increase in *pfpm2* copy number alone is not the sole modulator of PPQ resistance [31]. Silva et al. [32] have utilized gene editing and chemical inhibition to demonstrate that *pfpm2* amplification contributed to PPQ resistance and that the background of the engineered parasites was necessary to gain a bimodal dose–response, in which a second peak of survival for a subset of the parasite is detected at higher PPQ concentrations [22]. It was also suggested that the initial selection of *plasmepsin* and *pfmdr1* copy number variations developed a genetic background important for novel *pfcrt* mutations to emerge [32].

GWAS confirmed recrudescences from DHA–PPQ treatment failures identified a non-synonymous single nucleotide polymorphism (SNP) E415G substitution on an exonuclease encoding gene [20]. However, the role of E415G-Exo in mediating PPQ resistance, in the absence of other markers of resistance, is unclear. Specific novel mutations of the *pfcrt* gene have been shown to be associated with PPQ resistance since parasites with a variant of the Dd2 *pfcrt* allele, either T93S, H97Y, F145I, I218F, M343L, or G353V, have higher median PSA survival rates than those harboring the wildtype Dd2 allele [18, 26, 27], and a C350R substitution in the *pfcrt* gene resulted in decreased susceptibility to PPQ [33]. Dhingra et al. [27] showed that T93S and I218F-PfCRT mutations have increased in the past 5 years in Southeast Asia although showing an insignificant fitness cost compared to F145I-parasites.

To evaluate the proposed molecular markers of PPQ resistance associated with PPQ in vitro susceptibility, 17 parasite isolates were collected and successfully culture-adapted. Isolates were analysed for copy number variation of *pfmdr1*, *pfpm2* and SNPs of *pfkelch13, pfexo*, and *pfcrt*, and correlated with survival assays and drug susceptibility phenotypes. In addition, drug combination assays were utilized to complete isobolographic analysis on circulating *P. falciparum* parasites and to establish in vitro values and susceptibilities for various drug combinations.

## Methods

### Culture adaptation and maintenance of Cambodian parasites

The *P. falciparum* samples were collected from areas of documented multidrug resistance in Cambodia (Clinicaltrials.gov NCT02297477) [34]. Culture adaptation of the parasites was performed by thawing cryopreserved material containing infected red blood cells that had been mixed with glycerol mixture solution. Parasites were maintained in fresh human erythrocytes (O+) in RPMI-1640 medium (Sigma), containing 15% AB+ human serum (heat inactivated and pooled) and supplemented with 25 mM HEPES, 25 mM sodium bicarbonate, and 0.1 mg/mL gentamycin. Human blood products (erythrocytes and serum) were obtained from the Thai Red Cross. Cultures were placed in modular incubator chambers and gassed with 5% CO_2_, 5% O_2_, 90% N_2_ gas and incubated at 37 °C.

### Drug resistance genotyping

Parasite genomic DNA were extracted from 200 µL of whole blood using EZ1 DNA blood kit with automated EZ1 Advanced XL purification system or DNeasy^®^ Blood and Tissue (QIAGEN, Valencia, CA, USA) as per the manufacturer’s instructions and stored at − 20 °C. T100TM Thermal Cycler (Bio-Rad Laboratories) was employed to evaluate the propeller domain of the *P. falciparum kelch13* (*pfkelch13*) (amino acid residues 442–727) [12, 35], and *P. falciparum* exonuclease (*pfexo*) SNP at a codon corresponding to amino acid position 415 [20], while Master Cycler Nexus Gradient (Eppendorf) was used to detect the *pfcrt* SNP at codon corresponding to amino acid positions 93, 97, 145, 218, 343, 350, and 353 [26, 33]. Primers used to identify *pfkelch13*, *pfexo*, and *pfcrt* SNPs are shown in Additional file 1: Table S1. *P. falciparum* reference DNAs from 3D7 and W2 clones (Malaria Research & Reference Reagent Resource, Manassas, VA) were used as positive controls, and all samples were performed in duplicate.

### Copy number variation assay

To determine copy numbers of *pfmdr1* and *pfpm2* gene, real-time quantitative PCR (qPCR) was performed on genomic DNA as previously described [21, 36, 37] with some modifications. For *pfmdr1*, amplification reactions were performed according to the TaqMan Real-time PCR methods using ABI 7500 Real-time PCR system (Applied Biosystems) with 200 nM of each forward and reverse primer (Additional file 2: Table S2) and 2 ng of DNA template while Rotor-Gene Q (QIAGEN, Valencia, CA) using Type-it^®^ HRM™ kit was employed for *pfpm2* [21]. The primers used were as previously described to amplify the following loci: *pfmdr1* (PF3D7_0523000) and *pfpm2* (PF3D7_1408000), respectively. For the housekeeping gene, *β*-*tubulin* (PF3D7_1008700), β-tubulin forward and reverse primers were designed and used as a reference control for all experiments with the same validated PCR conditions as target primers. *Plasmodium falciparum* 3D7 and Dd2 were used as references for single and multiple copy number of *pfmdr1*, respectively. All samples including the references clones were performed in duplicate. The average copy number values for each genes were calculated using 2^−ΔΔCt^ method where ΔΔC_t_ is [C_t__*pfmdr1 or*__*pfpm2*_ − C_t__*pf β*-*tubulin*_] _sample_ − [C_t__*pfmdr1 or*__*pfpm2*_ − C_t__*pf β*-*tubulin*_] _3D7_. Parasites with copy number greater than 1.5 copies for *pfmdr1* [36] and 1.6 copies for *pfpm2* [21] were interpreted to contain multiple copies of each gene.

### In vitro 72-h drug susceptibility by Histidine rich protein 2 (HRP2)

Drug susceptibility test using HRP-2 ELISA to measure 50% or 90% inhibitory concentration (IC_50_ and IC_90_) was performed following previously published methods [17, 38]. Dried drug-coated plates containing antimalarial drugs dihydroartemisinin (DHA), artesunate (AS), mefloquine hydrochloride (MQ), quinine sulfate hydrate (QN), chloroquine diphosphate (CQ), lumefantrine (LUM), piperaquine tetraphosphate (PPQ), atovaquone (ATQ), doxycycline (DOX), artemisone (ATM), and cycloguanil (CYC) were prepared as described in Chaorattanakawee et al. [17, 39]. In vitro drug susceptibility testing was carried out for control reference clones (W2, D6, C2B) (Malaria Research & Reference Reagent Resource, Manassas, Vermont, USA), as described previously [39]. IC_50_s and IC_90_s were estimated by nonlinear regression analysis using GraphPad Prism version 6.0 program.

To determine a bimodal-dose response curve, the PPQ concentration (2.44 to 100,000 ng/mL) and the dilution series were increased from 8 to 24 points, according to the previously published report [22]. Culture-adapted clinical isolates were prepared in the similar manner as in in vitro drug susceptibility testing. The synchronized rings were grown for 72 h in the presence of different concentrations of PPQ (24-point dilution) in 96-well plates at 1.5% haematocrit, 0.5% starting parasitaemia in 0.5% Albumax RPMI 1640. Growth at 72 h was measured by HRP-2 ELISA. Assays were carried out in three biological replicates and the control reference clone W2 was tested along with each culture-adapted clinical isolate.

### Ring-staged survival assay (RSA)

In vitro RSA_0–3 h_ was performed on 0–3 h post-invasion rings obtained from selected culture-adapted clinical isolates following published methods [16] with slight modifications. The in vitro RSA_0–3 h_ tests were carried out in sequence numeric order by the study team that was blinded to the results of molecular markers of drug resistance. Briefly, parasite cultures were tightly synchronized using 5% d-sorbitol and 75% Percoll to obtain 0 to 3-h post-invasion rings which were adjusted to 0.5–1% starting parasitaemia with a 2% haematocrit in culture media. (0.5% Albumax RPMI 1640 with 2.5% AB serum), and cultured in a 48-well microplate with 700 nM DHA and 0.1% DMSO in separate wells for growth control. The culture plate was then incubated for 6 h at 37 °C in modular incubator chambers and gassed with 5% CO_2_, 5% O_2_ and 90% N_2_ gas. Cells were then washed once, resuspended in drug-free medium, and cultured further for 66 h. Susceptibility to DHA was assessed microscopically on thin films by estimating the percentage of viable parasites, relative to control (% survival rate). Parasites were counted from 10,000 red blood cells, and two separate individuals served as independent slide reader. In case of difference greater than 20%, slides were examined by a third microscopist blinded to the results. For the controls, the RSA_0–3 h_ was also performed on *P. falciparum* reference clones W2 (ART-sensitive control), IPC-4884 and IPC-5202 (BEI Resources, NIAID, NIH, USA) as ART-resistant control lines with reported % RSA survival value of 6.2% and 88.2%, respectively [16]. A survival rate > 1% was deemed resistant for RSA.

### Piperaquine survival assay (PSA)

PSAs were performed on culture-adapted clinical isolates with 0–3 h ring-stage parasite cultures following a previously published method [18] in sequence numeric order by the study team that was blinded to the results of molecular markers. Briefly, parasite cultures were tightly synchronized using 5% d-sorbitol and 75% Percoll to obtain 0 to 3-h post-invasion. Synchronized ring parasites at 0.5–1% starting parasitaemia and 2% haematocrit were incubated with 200 nM PPQ or 0.5% lactic acid in water at 37 °C for 48 h in a 48-well microplate. The cultures were then washed once, re-suspended in drug-free medium, and cultured further for 24 h. Susceptibility to PPQ was assessed microscopically on thin films by estimating the percentage of viable parasites in the similar manner as RSA. A survival > 10% was deemed resistant to PPQ.

### In vitro cloning of Cambodian *P. falciparum*

Parasite clones were obtained by the combination of limiting dilution cloning and plaque assay by plating a calculated 0.3 parasite per well in flat-bottomed 96-well microplate wells as described [40]. Wells containing single plaques were subsequently expanded into round-bottomed wells. The parasite clones were genotypically and phenotypically characterized. Once established, all clones were maintained in medium without any drug.

### Drug combination assay

Fixed ratio combinations of various antimalarial drugs were performed as previously described with some modifications [41, 42]. Stock solutions of the drugs were prepared at 1 mg/mL in 70% ethanol for DHA, MQ, CQ, tafenoquine (TQ), proguanil (PG), and pyronaridine (PND), in 0.5% lactic acid for PPQ, and in dimethyl sulfoxide for ATQ. The solutions for each drug were combined in ratios of 1 + 1, 1 + 3, 3 + 1, 1 + 4, 4 + 1, and 1 + 2; with each drug also tested alone. 50 µL of single and combination drug solutions were then introduced into 96-well plates to give a row with two-fold serial dilutions. 200 µL of parasite culture with a final parasitaemia of 0.5% in a 2% haematocrit were added, and the test plates were incubated for 72 h at 37°C in modular incubator chambers and gassed with 5% CO_2_, 5% O_2_ and 90% N_2_ gas. Parasite growth was measured by HRP2 drug susceptibility testing as described above. The individual 50% fractional inhibitory concentrations (FIC_50_) were determined as previously described [43]. Isobolograms were constructed by plotting the FIC_50_ of drug A against the FIC_50_ of drug B for each of the six drug ratios. A concave curve indicated synergy, a straight line represented additivity and a convex curve indicated antagonism. To obtain numeric values for the interaction, results were expressed as the sum of the FIC_50A_ and FIC_50B_. The sum FIC_50_ (ΣFIC_50_) values indicate the kinds of interaction as follows: synergism when ΣFIC_50_ ≤ 0.5; toward synergism when ΣFIC_50_ < 1; additive when ΣFIC_50_ = 1; toward antagonism when ΣFIC_50_ > 1; antagonism when ΣFIC_50_ ≥ 2 to 4. The IC_50_s of each drug in the test combination were standardized by allocating the value of 1 to each drug that was tested alone and prorated values for each fixed concentration ratio.

### Statistical analysis

Statistical analysis was performed using GraphPad Prism version 6.0 (GraphPad Software, Inc., San Diego, CA, USA). In vitro parasite susceptibility to each test drug was expressed as mean IC_50_s for all samples. The difference of the IC_50_ values between groups was assessed by nonparametric Kruskal–Wallis, Mann–Whitney or Dunn’s multiple comparison tests, as appropriate. Statistical significance was defined as a *p* value of < 0.05.

## Results

### Molecular genotypes of culture-adapted clinical isolates

Seventeen *P. falciparum* clinical isolates were successfully culture-adapted and underwent genotypic profiling. The parasite isolates were classified into 4 groups based on the molecular markers. Group 1, parasite isolates 14 and 17, contained K13-, Exo- and PfCRT-wild-type with a single copy of *pfpm2*. Group 2, parasite isolates 13, harboured C580Y-K13 mutation, Exo- and PfCRT-wild-type with a single copy of *pfpm2*. Group 3, parasite isolates 4 and 9, had C580Y-K13, E415G-Exo, and PfCRT mutations with a single copy of *pfpm2.* Group 4, parasite isolates 1, 2, 3, 5, 6, 7, 8, 10, 11, 12, 15, and 16, carried C580Y-K13, E415G-Exo, and PfCRT mutations with multiple copies of *pfpm2* (Table 1).

**Table 1:** Molecular genotyping of in vitro parasite samples

Sample ID	*pfmdr1* CN	K13 C580Y	*pfpm2* CN	Exo E415G	PfCRT							Group
					T93S	H97Y	F145I	I218F	M343L	C350R	G353V	
1	0.72	*Y*	*2.85*	*G*	T	*Y*	F	I	M	C	G	4
2	0.96	*Y*	*1.80*	*G*	T	H	*I*	I	M	C	G	4
3	0.88	*Y*	*2.13*	*G*	T	H	*I*	I	M	C	G	4
4	0.90	*Y*	0.84	*G*	T	H	F	*F*	M	C	G	3
5	0.91	*Y*	*2.39*	*G*	T	H	F	*F*	M	C	G	4
6	0.89	*Y*	*1.83*	*G*	T	H	*I*	I	M	C	G	4
7	0.84	*Y*	*1.85*	*G*	T	H	F	*F*	M	C	G	4
8	0.89	*Y*	*2.73*	*G*	T	H	*I*	I	M	C	G	4
9	0.93	*Y*	1.48	*G*	T	H	*I*	I	M	C	G	3
10	0.96	*Y*	*2.05*	*G*	T	H	*I*	I	M	C	G	4
11	0.78	*Y*	*2.22*	*G*	*S*	H	F	I	M	C	G	4
12	0.80	*Y*	*2.35*	*G*	T	H	F	I	M	C	G	4
13	0.89	*Y*	1.00	E	T	H	F	I	M	C	G	2
14	0.84	C	1.04	E	T	H	F	I	M	C	G	1
15	0.80	*Y*	*2.71*	*G*	T	H	F	*F*	M	C	G	4
16	0.82	*Y*	*2.48*	*G*	T	H	F	*F*	M	C	G	4
17	0.66	C	1.08	E	T	H	F	I	M	C	G	1

Italic indicates either mutations or multiple copy number. A cut-off copy number of 1.5 and 1.6 are used to define *pfmdr1*and *pfpm*2 multiple copy number. Group 1 is for parasites containing WT-K13, WT-Exo, WT-PfCRT, and single copy number (CN) *pfpm2*. Group 2 is for parasites containing C580Y-K13, WT-Exo, WT-PfCRT, and single CN *pfpm2*. Group 3 is for parasites harboring C580Y-K13, E415G-Exo, Mut-PfCRT, and single CN *pfpm2*. Group 4 is for parasites having C580Y-K13, E415G-Exo, Mut-PfCRT, and multiple CN *pfpm2*

### Correlation of ART- and PPQ-resistance markers with RSA and PSA phenotypes

**Fig. 1:**
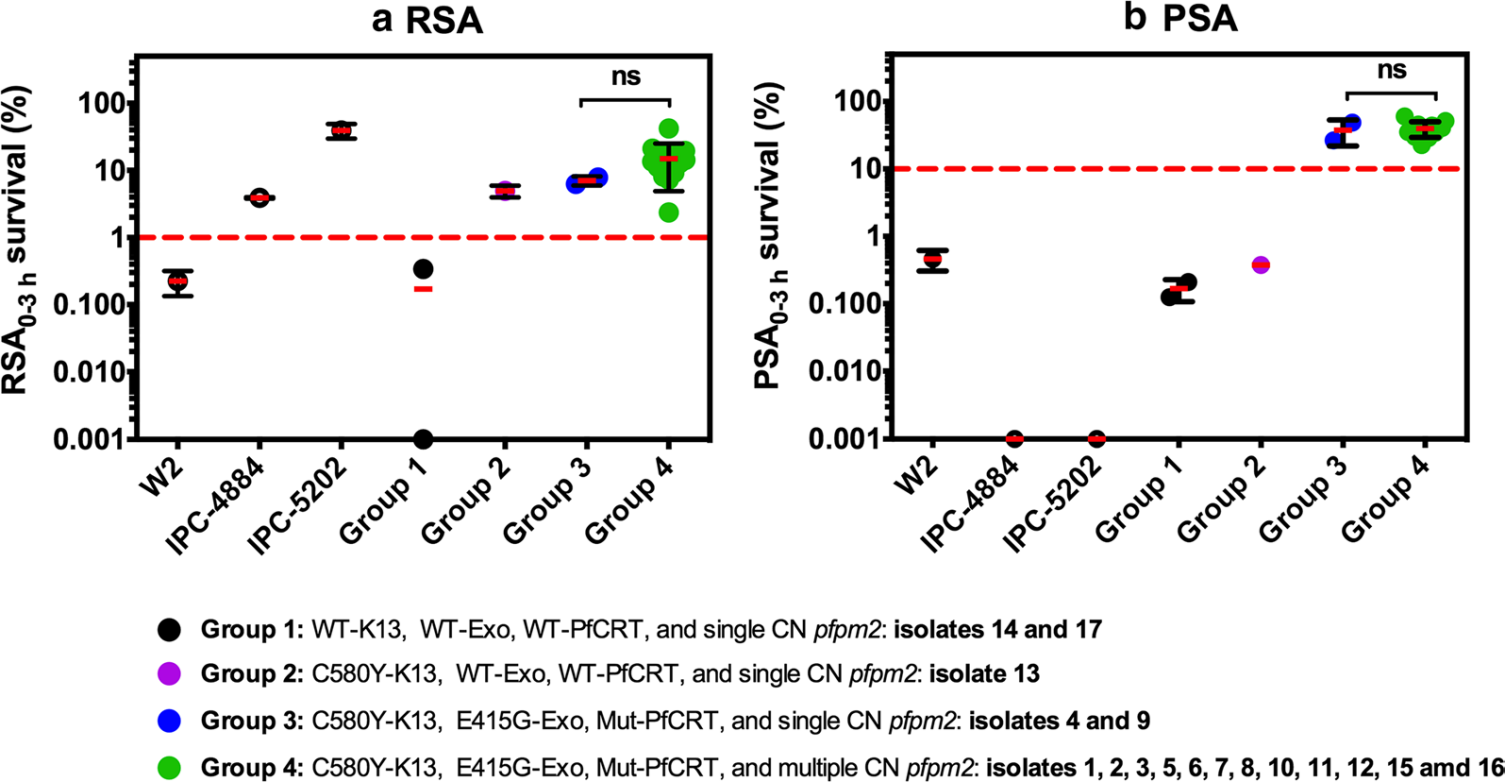
Survival assay for ART- and PPQ-resistance. **a** In vitro RSA_0-3h_ survival rates for standard laboratory-adapted clones (W2 for an ART-sensitive control, IPC-4884 and IPC-5202 for ART-resistance control) and culture-adapted clinical isolates. The dashed line represents the 1% survival rate cut-off that differentiates ART-resistance (≥ 1%, red-dashed line) from ART-sensitive (< 1%) parasites in RSAs. **b** In vitro PPQ survival assay (PSA_0–3 h_) survival rates for standard laboratory-adapted clones (W2, IPC-4884 and IPC-5202 for PPQ-sensitive parasites) and culture-adapted clinical isolates. The dashed line represent the 10% survival rate cut-off that distinguishes PPQ-resistance (≥ 10%) from PPQ-sensitive (< 10%, red dashed line) parasites in PSAs. Two biological replicates were performed and survival rates are presented as mean ± S.D. Significance was determined using Mann–Whitney U test. Group 3 and Group 4 are compared. ns is not significant (p ≥ 0.05). Zero values of % survival rate were plotted as 0.001% in logarithmic scale

The phenotypic analysis was performed to determine whether the molecular markers were associated with ART- and PPQ-resistance. Parasites without the C580Y-K13 mutation (Group 1) exhibited % RSA survival rate of less than 1 (a cut-off for ART resistance), while parasites with the C580Y-K13 mutation (Group 2–-4) all had % survival rate of greater than 1, clearly demonstrating the correlation between the C580Y-K13 marker and ART resistance (Fig. 1a, Additional file 3: Table S3).

The relationship between *pfpm2* copy number (CN), the E415G-Exo and novel PfCRT mutations and the % PSA survival of the parasites was assessed (Fig. 1b, Additional file 3: Table S3). PPQ-sensitive parasites *P. falciparum* W2, IPC-4884, and IPC-5202 were used as controls. Parasites in Group 1 and 2, lacking PPQ-resistance markers (*pfpm2* multiple CN, the E415G-Exo and novel PfCRT mutations) showed % PSA survival of less than 10, indicative of PPQ-sensitivity. Parasites from Group 3 and 4, which contained E415G-Exo and PfCRT mutations, exhibited % PSA survival higher than 10, indicative of PPQ-resistance. Although parasites from group 3 had a single copy of *pfpm2* and parasites from group 4 had multiple copies of *pfpm2*, there were no observed differences in % PSA survival between these two groups.

### In vitro drug susceptibility and cell morphology of culture-adapted clinical isolates

**Fig. 2:**
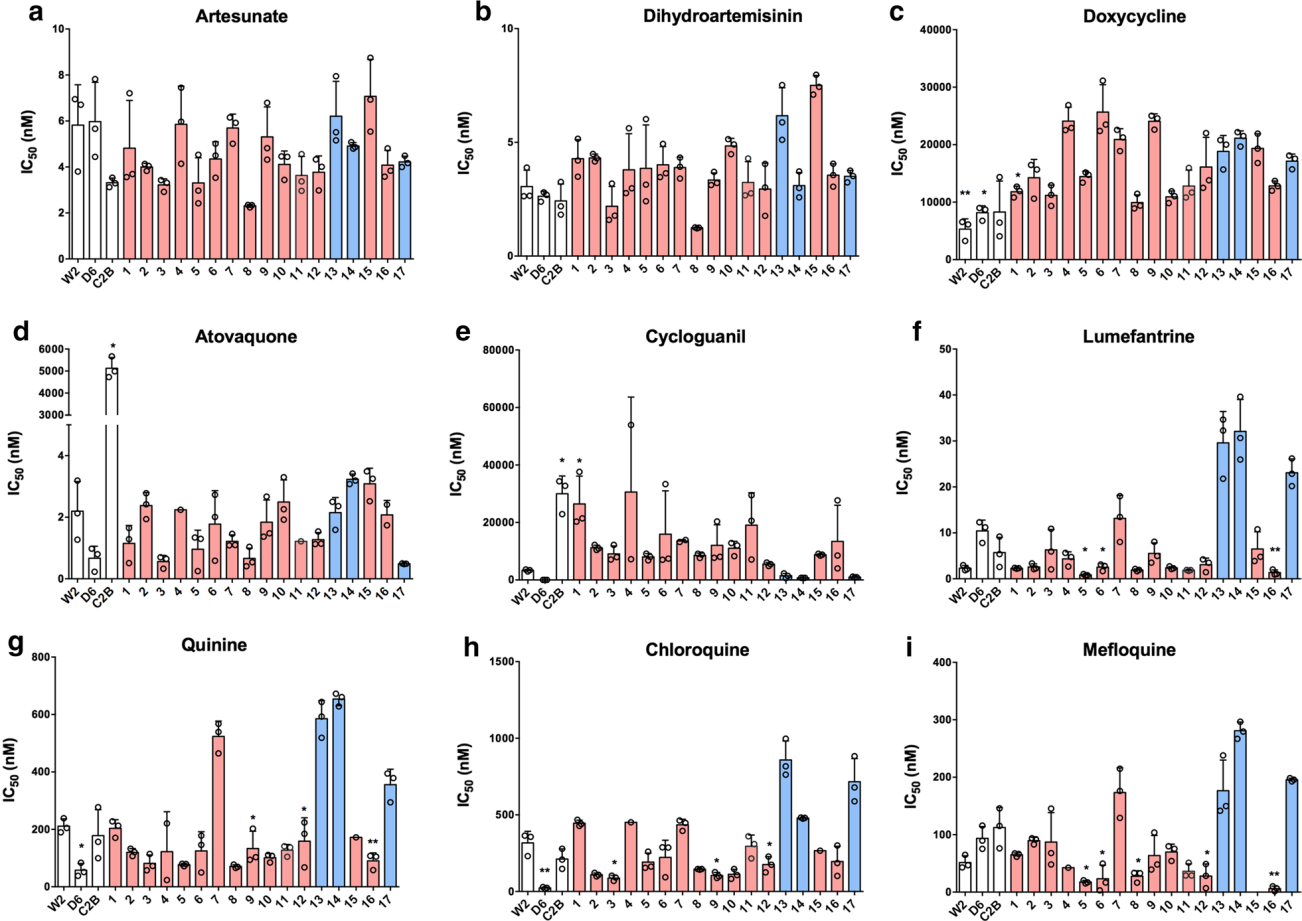
In vitro *P. falciparum* susceptibility to multiple antimalarial drugs. Mean ± S.D IC_50_ values were calculated from 72-h dose–response assays for drugs designated in **a**–**i**. White bars represent standard laboratory-adapted clones, while blue and red bars indicate clinical-adapted parasites with PPQ sensitive or PPQ resistance, respectively. Three biological replicates were carried out for each sample. Statistically significant differences relative to isolate 17 are indicated with one (0.05 > p > 0.01) and two (p < 0.01) asterisks

**Fig. 3:**
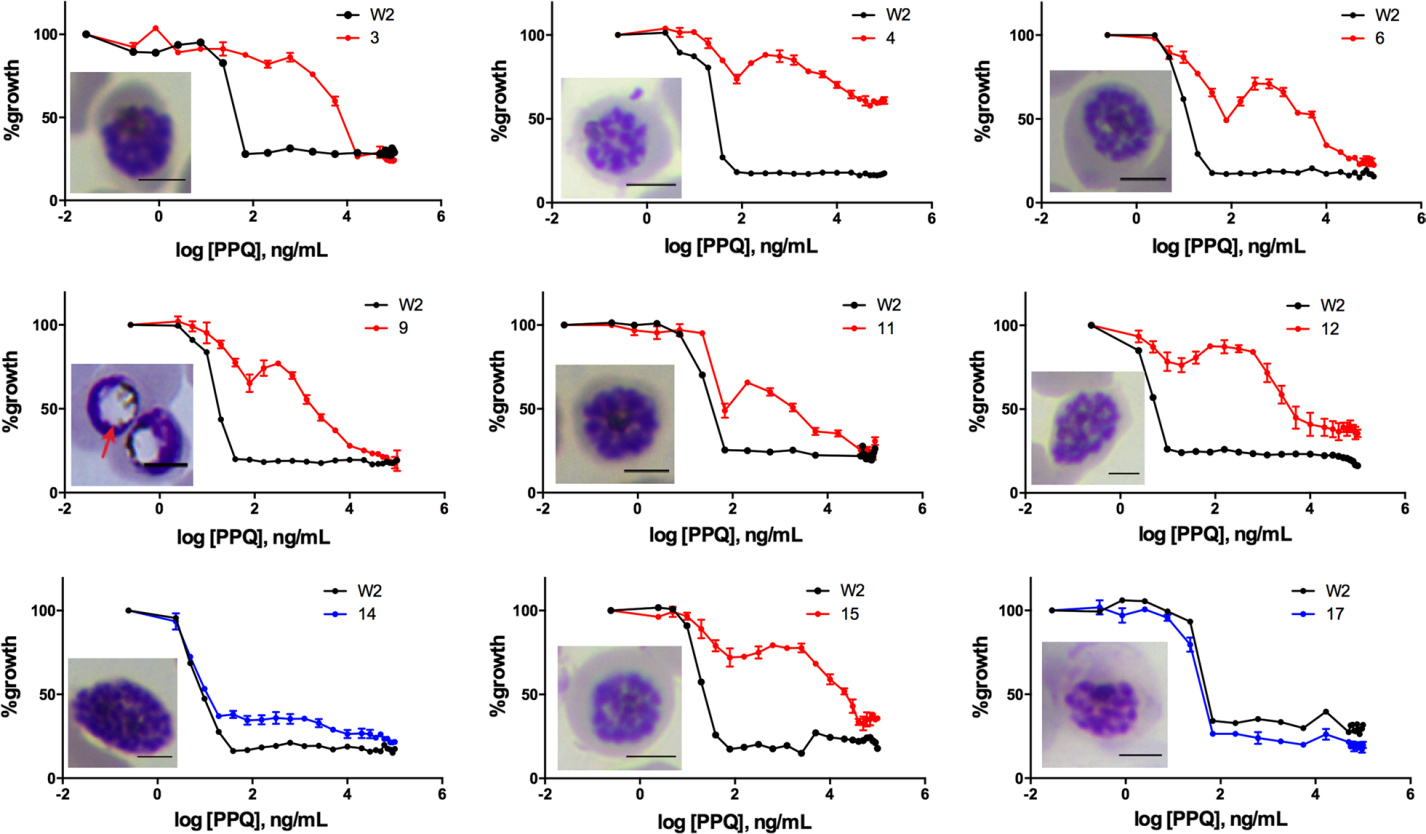
PPQ dose–response curves and cell morphology for selected culture-adapted clinical isolates. Increasing the starting concentration and number of data points (24 points) for HRP2 ELISA dose–response curve provided a bimodal distribution of parasite response to PPQ exposure for PPQ-resistant parasites. The PPQ-sensitive W2 parasite (black line) is shown alongside the culture-adapted clinical isolates. The PPQ-sensitive clinical adapted parasites are shown in blue lines, whereas the PPQ-resistant clinical adapted parasites are represented in red lines. Data are shown as mean values from three biological replicates with S.D for isolates. Cell morphology of selected culture-adapted clinical isolates shows that isolate number 9 revealed distended, translucent DV (the red arrow) similar to the engineered parasite with PfCRT variants [26]. The scale bar, 50 µm

To get a better understanding of cross-resistance, the parasites were tested against a panel of antimalarial drugs (Fig. 2 and Additional file 4: Table S4). The samples were categorized into PPQ-resistant (PPQ-R) (red bars, Groups 3 and 4) and PPQ-sensitive (PPQ-S) (blue bars, Groups 1 and 2) isolates. PPQ-S and PPQ-R parasites had similar IC_50_ values for AS, DHA, DOX, and ATQ. However, the PPQ-R parasites had higher IC_50_ for CYC and lower IC_50_ values for LUM, QN, CQ, and MQ. This suggests reciprocal drug resistant pattern for PPQ and the following drugs, LUM, QN, CQ, and MQ. To assess whether the PPQ resistant field isolates exhibited a second peak of survival around 0.1–10 μM (Bimodal dose–response) as was shown by Bopp et al. [22] in Cambodian parasites, the starting PPQ concentration was increased from 0.5 to 50 μM and the dilution series extended from 12 to 24 points (Fig. 3). PPQ-R parasites exhibited the second peak of survival around 78–20,000 ng/ml (or 0.08–21 µM), indicating a bimodal dose–response curve. Unlike PPQ-R parasites, PPQ-S parasites did not show the second peak, and their dose–response curves were similar to that of the reference clone W2.

Ross et al. [26] has shown previously that *pfcrt*-edited Dd2 parasites developed a distended and translucent DV phenotype during the development from mid-trophozoites to mid-schizonts. This trait was specific to the *pfcrt*-edited Dd2 with F145I-, M343L- and G353V-PfCRT mutations and a single copy of *pfpm2* but not observed in the PPQ-resistant Cambodian lines PH1008-C (multiple copies of *pfpm2* and M343L-PfCRT) or PH1263-C (multiple copies of *pfpm2* and H97Y-PfCRT) [26]. To validate if this phenotype could be observed with PPQ resistant parasites collected from the field, schizont morphology was examined. Among the parasites analysed only isolate 9 exhibited the swollen and translucent DV (Fig. 3). This isolate contained F145I-PfCRT and E415G-Exo mutations but a single copy of *pfpm2*. Parasite isolate 4 had a similar pattern of PPQ-resistance markers (except I218F-PfCRT instead of F145I-PfCRT) but no distended DV was observed. Parasites carrying both an F145I-PfCRT mutation and multiple copies of *pfpm2* (such as parasite isolates 3 and 6) did not show an altered DV morphology. This is similar to the observation found in the PPQ-resistant Cambodian lines PH1008-C and PH1263-C [26].

### Cloning of Cambodian *P. falciparum* isolate

Clinical isolates of *P. falciparum* are a genetically heterogeneous population of parasites. To obtain stable strains of the parasites for long term experiments, a rapid method of cloning was developed using a combination of limiting dilution and plaque assay [40]. Several attempts were carried out to clone 8 Cambodian *P. falciparum* isolates (isolates 3, 4, 6, 9, 12, 14, 15, and 17) and while all of the selected samples generated a single plaque after 7 days, only clones from isolate 14 could be expanded. After 1 month, four clones from isolate 14 were established including 14-B5, 14-C6, 14-C7, and 14-F5. These clones were genotypically and phenotypically characterized and compared to parent isolate and standard lab clones *P. falciparum* 3D7 and W2 (Additional file 5: Figure S1). All 4 clones possessed the identical genotypes to the parent isolate, and the RSA and PSA survival assays reflected sensitivity to ART and PPQ (Additional file 5: Figure S1).

**Fig. 4:**
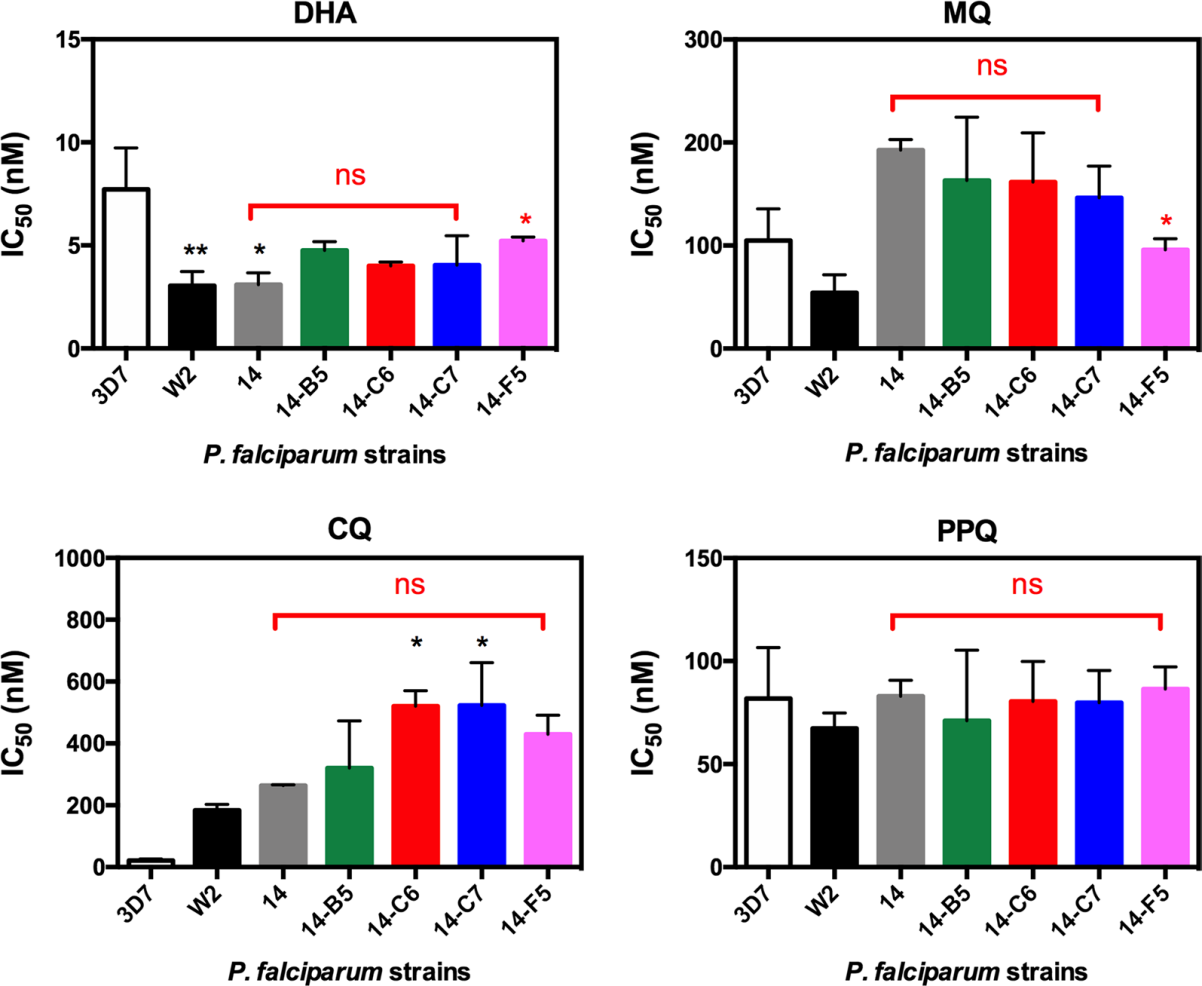
Drug susceptibility of *P. falciparum* Cambodian isolate 14 before and after cloning against DHA, MQ, CQ, and PPQ. The W2 and 3D7 strains of *P. falciparum* were used as controls. Statistically significant differences relative to 3D7 and isolate 14 are indicated in black and red asterisks, respectively with 0.05 > p > 0.01 for one asterisk and p < 0.01 for two asterisks. Abbreviation, ns is for not significant difference (p ≥ 0.05)

Drug susceptibility profiles of the clones and parent isolate are illustrated in Fig. 4 and Additional file 6: Table S5. Compared to 3D7 (CQ-sensitive), all clones and the parent isolate exhibited high IC_50_s toward CQ similar to that of W2 (CQ-resistance). MQ IC_50_s for the clones were much higher than for W2 isolate (MQ-sensitive), and comparable to IC_50_ of D6 (MQ-resistance, IC_50_-MQ = 130.8 ± 15.96 nM). Collectively, based on the drug susceptibility profile and survival assays all four clones (14-B5, 14-C6, 14-C7, and 14-F5) were classified as ART- and PPQ-sensitive, but CQ-resistant and having reduced MQ sensitivity.

### Drug combination testing of *P. falciparum* 14-B5

Since a new clone from parasite field isolates was successfully obtained, this clone was evaluated for sensitivity against a diverse array of drug combinations utilizing the HRPII-ELISA to establish synergistic, additive, and antagonistic in vitro anti-malarial drug interactions. As validation controls, the fixed ratio combinations of DHA–PPQ, CQ–CQ, and ATQ–PG were first tested against *P. falciparum* 3D7, W2, D6, C2B, and IPC-5202 strains as well as 14-5B clone. Table 2 and Additional file 7: Table S6 represent summary of drug interaction and the ΣFIC_50_s of tested fixed drug ratio combination, respectively. DHA–PPQ revealed antagonistic interactions, as reported previously [44]. CQ–CQ, serving as an experimental drug combination control, showed the additive interaction while ATQ–PG revealed a synergistic interaction as previously reported by Co et al. [42].

**Table 2:** Summary of drug interaction in asexual stages of different *P. falciparum* strains

Drug Combination	*P. falciparum* strain					
	3D7	W2	D6	C2B	IPC-5202	14-B5
DHA–PPQ	Antagonistic	Antagonistic	Antagonistic	Antagonistic	Antagonistic	Antagonistic
CQ–CQ	Additive	Additive	Additive	Additive	Additive	Additive
ATQ–PG	Synergistic	Synergistic	Synergistic	Synergistic	Synergistic	Synergistic
PND–ATQ	Antagonistic	Antagonistic	Antagonistic	Toward Antagonistic	Antagonistic	Antagonistic
MQ–ATQ	Toward Antagonistic	Antagonistic	Toward Antagonistic	Toward Synergistic	Toward Antagonistic	Antagonistic
TQ–ATQ	Toward Synergistic	Toward Antagonistic	Toward Synergistic	Toward Synergistic	Toward Antagonistic	Antagonistic
PND–PG	Toward Antagonistic	Toward Synergistic	Additive	Toward Synergistic	Toward Synergistic	Antagonistic
MQ–PG	Toward Synergistic	Toward Synergistic	Toward Synergistic	Toward Synergistic	Toward Synergistic	Toward Synergistic
TQ–PG	Synergistic	Toward Antagonistic	Toward Synergistic	Toward Synergistic	Additive	Antagonistic

ΣFIC_50_ (50% Fractional Inhibitory Concentrations), synergism when ΣFIC_50_ ≤ 0.5; toward synergism when ΣFIC_50_ < 1; additive when ΣFIC_50_ = 1; toward antagonism when ΣFIC_50_ > 1; antagonism when ΣFIC_50_ ≥ 2 to 4. The values show the mean ± S.D of 3 independent assays for each parasite line

*DHA* dihydroartemisinin, *CQ* chloroquine, *MQ* mefloquine, *PPQ* piperaquine, *ATQ* atovaquone, *PG* proguanil, *TQ* tafenoquine, *PND* pyronaridine

Potential novel combinations with either ATQ or PG were tested. When PND or MQ were combined with ATQ, antagonism/toward antagonism, except for MQ–ATQ in the C2B strain (Table 2) was observed. In TQ–ATQ combination, responses varied across strains although only one line, 14-B5 clone, revealed antagonistic interaction. When combined with PG, both PND and TQ showed different responses across strains, with 14-B5 showed antagonism between TQ–PG. All of the tested parasites revealed toward synergistic drug interaction against MQ–PG combination.

## Discussion

In this study, the in vitro drug sensitivity and molecular profiles of *P. falciparum* isolates that were collected in Cambodia, a location well known as the epicenter of drug resistance are reported. The samples were collected at the time when PPQ resistance has been widespread. The CQ and ART resistant strains of *P. falciparum* likely originated in Cambodia, not far from location where these field isolates were collected, thus having unique genetic background. Twenty-six isolates were aimed to in vitro culture but only 17 isolates could be successfully cultured. Most of the analysed samples contained C580Y-K13 mutations and were labelled as ART-R based on RSA results. There may be more than a single pathway to ART resistance with other markers of resistance [45–47]; nonetheless the C580Y-K13 mutation is entrenched in this region consistent with observations for the field isolates included in this analysis. In addition, based on molecular markers and in vitro PSA testing, 14 of 17 *P. falciparum* samples were piperaquine-resistant. Isolates with all three PPQ resistance markers: amplification of *pfpm2,* presence of E415G-Exo and PfCRT mutations (T93S, H97Y, F145I, and I218F) showed a decrease in PPQ sensitivity as was expected. However, parasites carrying only the E415G-Exo and PfCRT mutations also resulted in PPQ resistant phenotype, without the requirement for *pfpm2* amplification as was previously reported in the literature [20–22]. While increased *pfpm2* copy number is accepted as a marker of PPQ resistance [20, 22, 24, 48, 49], molecular surveillance for PPQ resistance should not rely solely on the *pfpm2* copy number amplification, as this may result in PPQ resistance being undetected in areas where PPQ resistance may be driven by other mutations, involving E415G-Exo and PfCRT mutations, in the absence of *pfpm2* amplification. Since multiple copies of *pfpm2* are commonly found in concert with PfCRT mutations, it has been suggested that *pfpm2* amplification might help overcome the fitness cost of PfCRT variants by increasing the rate of hemoglobin degradation, and quick sequestration of reactive haem into haemozoin [33] and therefore improving the likelihood of PPQ resistant strains being maintained in the population. All analysed parasites held a single copy of *pfmdr1,* indicating that this variant should not be used as PPQ resistance marker. The treatment with DHA–PPQ was common when the field isolates were collected and most likely resulted in low MQ pressure, thereby promoting the loss of *pfmdr1* amplifications in the parasites [20]. However, the limitation of this analysis is the lack of direct attribution of clinical outcome to the presence of molecular markers and in vitro drug sensitivity data.

Bimodal dose–response curves documented from Cambodian parasites collected in 2011 were also observed in the PPQ-R parasites consistent with reports by Bopp et al. [22]. The results suggested that bimodal dose–response curves can be observed in all PPQ resistant parasites regardless of PPQ resistance markers and the presence of the secondary peak in dose response curve might be another indicator of PPQ-R and was confirmed to be present in the field isolates. It has also been demonstrated that *P. falciparum* lines with PfCRT mutations in (C101F or L272F) result in the swelling of the parasite’s food vacuole and increased susceptibility to chloroquine and other quinoline antimalarials [50]. The mutations in PfCRT may interfere with the transport of the natural substrates out of the food vacuole, resulting in increased osmotic pressure. This phenotype was also observed in Dd2 parasites expressing the PfCRT mutations F145I, M343L, and G353V, [26]. In this study, the parasite isolate 9 which contained PfCRT-F145I mutation and *pfpm2* single copy exhibited a swollen and translucent DV phenotype, while parasite isolates 3 and 6, that had PfCRT mutations and multiple copies of *pfpm2*, did not show the characteristic of swollen DVs. On the other hand, parasite isolate 4 containing I218F-PfCRT mutation and single copy *pfpm2* did not show a swollen DV. This result might imply that not all novel PfCRT mutations exhibit a swollen DV phenotype, depending on the location of the mutated amino acids. Clinical implication and causative mechanisms of this finding still remain to be elucidated.

PPQ-resistant parasites were shown to be more susceptible to QN, CQ, MQ and LUM, similar to the engineered parasites expressing the novel PfCRT mutations [26]. This may have enabled a successful switch of first-line treatment from DHA–PPQ to artesunate–mefloquine by the Cambodian government in 2016. However, in this study, the IC_50_s against CYC seemed to be higher in PPQ resistant isolates (mean values ranging from 5470 nM to 30,585 nM) than in PPQ sensitive isolates (mean values ranging from 716 to 1435 nM). Overall, parasites demonstrated high level CYC resistance (geometric mean pretreatment ex vivo IC_50_, 2204 nM) [34]. PPQ-R isolates from Cambodia had greater susceptibility to LUM which may be a promising drug combination that should be explored in future studies. Further study is required to understand the implications of increased IC_50_ of CYC in the PPQ resistant parasites.

With the current decline of efficacy of partner drugs in the available artemisinin-based combinations, there is pressing need to evaluate novel compounds and new anti-malarial combinations against currently circulating field isolates. By culture-adapting field isolates, four clones (14-B5, 14-C6, 14-C7, and 14-F5) were obtained, and can be used as *P. falciparum* lines for HRP2-ELISA drug combination susceptibility testing to help quantify the contribution of different drug components on risk of treatment failure. Presented data confirmed findings by others who used the SYBR green I-based fluorescence (MSF) assay [42] and [^3^H]-hypoxanthine incorporation method [44] to demonstrate a synergistic interaction for ATQ–PG, an additive effect for CQ–CQ, and an antagonistic interaction for DHA–PPQ. It is interesting to observe that the 14-B5 clone tracks very similar to W2 against every tested drug combination except for PND–PG in which W2 is towards synergistic but 14-B5 is antagonistic. Of the drug combination tested, MQ–PG combination provided at least mild synergistic interactions against all the tested parasites including the 14-B5 line, where other drug combinations displayed antagonism. These findings demonstrate the importance of using currently circulating parasite isolates for evaluating the drug combinations as responses may be different based on unique genetic backgrounds. The diversity of parasites and variations in drug interactions that were observed among isolates may provide new insights into the outcomes of clinical studies in Cambodia.

## Conclusion

The assessment of molecular markers associated with ART and PPQ resistance provides valuable information on how parasites have responded to DHA–PPQ exposure. This study shows that *P. falciparum* harbouring either *pfpm2* amplification or novel PfCRT mutations with E415G-exo mutation display PPQ resistant phenotype. The increased copy number of *pfpm2* may not be required for PPQ resistant phenotype. The cloned Cambodia parasite exhibit varied sensitivities to in vitro drug combinations. Genetic background of circulating field isolates is important for assessment of drug combinations and the use of the latest field isolates may be necessary for assessment of relevant drug combinations against *P. falciparum* resistant strains.

## Supplementary information

**Additional file 1: Table S1.** The oligonucleotides of each primers *P. falciparum* for drug-resistant markers testing, including *pfkelch13* (covering amino acid positions 539 to 580), *pfexo* (amino acid position 415), and *pfcrt* (at amino acid positions 93, 97, 145, 218, 343, 350, and 353) performed by T100™ Thermal Cycler.

**Additional file 2: Table S2.** The oligonucleotides of each primers for copy number variation of *P. falciparum* multidrug resistance gene 1 (*pfmdr1*) and *P. falciparum* plasmepsin 2 *(pfpm2)* with β-tubulin, the reference gene, performed by ABI 7500 Real-time PCR system.

**Additional file 3: Table S3.** Mean ± S.D of  % Survival rate of Ring-stage Survival Assay (RSA) and Piperaquine Survival Assay (PSA).

**Additional file 4: Table S4.** IC_50_s of asexual stage *P. falciparum* parasites for different antimalarial drugs (mean ± S.D). N/A is not-applicable.

**Additional file 5: Figure S1.** Characterization of a new field clone of *P. falciparum* isolate. (A) Sequencing chromatogram of *pfexo* gene confirmed the amino acid at position 145 of Exonuclease from *P. falciparum* Cambodian isolate 14 is glutamic acid (E), which is coded by a codon GAG. (B) Table represents molecular genotyping of *P. falciparum* Cambodian isolate 14 before and after cloning. The copy number of *pfmdr1* and *pfpm2* less than 1.5 and 1.6 indicates a single copy of the gene, respectively, while  % RSA and  % PSA cut off less than 1 and 10 represents the ART- and PPQ-sensitive, respectively. N.D stands for not determined.

**Additional file 6: Table S5.** Comparison of the in vitro susceptibility of *Plasmodium falciparum* Cambodian isolate 14 before and after cloning as well as *P. falciparum* 3D7 and W2 exposed to established antimalarial agents. IC_50_ values report the mean ± S.D from at least 3 experiments. Statistically significant difference relative to 3D7 is indicated with one asterisk (0.05 > p > 0.01). N/A is non-applicable.

**Additional file 7: Table S6.** In vitro drug combination assay for 9 combinations in asexual stages of different *P. falciparum* strains. DHA, dihydroartemisinin; CQ, chloroquine; MQ, mefloquine; PPQ, piperaquine; ATQ, atovaquone; PG, proguanil; TQ, tafenoquine; PND, pyronaridine. ΣFIC_50_ (50% Fractional Inhibitory Concentrations), synergism when ΣFIC_50_ ≤ 0.5; toward synergism when ΣFIC_50_ < 1; additive when ΣFIC_50_ = 1; toward antagonism when ΣFIC_50_ > 1; antagonism when ΣFIC_50_ ≥ 2 to 4. The values show the mean ± S.D of 3 independent assays for each parasite line.

## Data Availability

All data generated or analysed during this study are included in this published article and its supplementary information files.

## Abbreviations

ACT: Artemisinin-based combination therapy
AS: Artesunate
ATM: Artemisone
ATQ: Atovaquone
ART: Artemisinin
Cas: CRISPR associated protein
CN: Copy number
CQ: Chloroquine
CRISPR: Clustered regularly interspaced short palindromic repeats
CYC: Cycloguanil
DHA: Dihydroartemisinin
DHFR: Dihydrofolate reductase
DOX: Doxycycline
DV: Digestive vacuole
ELISA: Enzyme-linked immunosorbent assay
Exo: Exonuclease
FIC: Fractional inhibitory concentration
GWAS: Genome-wide association studies
HEPES: 4-(2-Hydroxyethyl)-1-piperazineethanesulfonic acid
HRP: Histidine rich protein
K13: Kelch-13
LUM: Lumefantrine
MQ: Mefloquine
PfCRT: *P. falciparum* chloroquine resistance transporter
Pfmdr1: *P. falciparum* multidrug resistance 1
PG: Proguanil
PM: Plasmepsin
PND: Pyronaridine
PPQ: Piperaquine
PQ: Primaquine
PSA: PPQ survival assay
QN: Quinine
qPCR: Quantitative real-time polymerase chain reaction
RPMI: Rosewell Park Memorial Institute
RSA: Ring-stage survival assay
SNP: Single nucleotide polymorphism
TQ: Tafenoquine
WHO: World Health Organization
