# Supplementary material for: Piperaquine resistant Cambodian Plasmodium falciparum clinical isolates: in vitro genotypic and phenotypic characterization
Source: Malar J. 2020 Jul 25;19:269. doi: 10.1186/s12936-020-03339-w (PMC7382038; doi:10.1186/s12936-020-03339-w)
Supplement: Supplementary file 1 — Additional file 1: Table S1. The oligonucleotides of each primers P. falciparum for drug-resistant markers testing, including pfkelch13 (covering amino acid positions 539 to 580), pfexo (amino acid position 415), and pfcrt (at amino acid positions 93, 97, 145, 218, 343, 350, and 353) performed by T100™ Thermal Cycler. [file 12936_2020_3339_MOESM1_ESM.docx]

**Table S1**.

| **Primer** | | **Sequence (5´ 🡪 3´)** | |
| --- | --- | --- | --- |
| *pfkelch13* | Outer Forward | | CGG AGT GAC CAA ATC TGG GA |
|  | Outer Reverse | | GGG AAT CTG GTG GTA ACA GC |
|  | Nested Forward | | GCC AAG CTG CCA TTC ATT TG |
|  | Nested Reverse | | GCC TTG TTG AAA GAA GCA GA |
| *pfexo-E415G*  *pfcrt-T93S-H97Y-F145I* | Forward | | GCA CCT CCT ATC ATC AGA TGA TAC C |
|  | Reverse | | CAA GAA AAG AGG AAG GAA CAC CTT C |
|  | Forward | | TGC TAA AAG AAC TTT AAA CAA AAT TGG |
|  | Reverse | | CAA GAA CTA CTG GAA ATA TCC AAT C |
| *pfcrt-I218F* | Forward | | TCT CGG AGC AGT TAT TAT TGT TG |
|  | Reverse | | ATT TCC CTT GTC ATG TTT GAA |
| *pfcrt-M343L-G353V* | Forward | | CGC ATT GTT TTC CTT CTT TAA C |
|  | Reverse | | CGG CTA AGA ATT TAA AGT AAT AAG CAA |
