# Supplementary material for: Piperaquine resistant Cambodian Plasmodium falciparum clinical isolates: in vitro genotypic and phenotypic characterization
Source: Malar J. 2020 Jul 25;19:269. doi: 10.1186/s12936-020-03339-w (PMC7382038; doi:10.1186/s12936-020-03339-w)
Supplement: Supplementary file 2 — Additional file 2: Table S2. The oligonucleotides of each primers for copy number variation of P. falciparum multidrug resistance gene 1 (pfmdr1) and P. falciparum plasmepsin 2 (pfpm2) with β-tubulin, the reference gene, performed by ABI 7500 Real-time PCR system. [file 12936_2020_3339_MOESM2_ESM.docx]

**Table S2**.

| **qPCR** | **Primer** | | **Sequence (5′ 🡪 3′)** | |
| --- | --- | --- | --- | --- |
| *pfmdr1* | *pfmdr1* Forward | TGC ATC TAT AAA ACG ATC AGA CAA A | | |
|  | *pfmdr1* Reverse | TCG TGT GTT CCA TGT GAC TGT | | |
|  | *pfmdr1-*probe | 6FAM-TTT AAT AAC CCT GAT CGA AAT GGA ACC TTT G-TAMRA | |  |
|  | β-*tubulin* Forward | AAA AAT ATG ATG TGC GCA AGT GA | |  |
|  | β-*tubulin* Reverse | AAC TTC CTT TGT GGA CAT TCT TCC T | |  |
|  | β-*tubulin-*probe | VIC-TAG CAC ATG CCG TTA AAT ATC TTC CAT GTC T-TAMRA | |  |
| *pfpm2* | *pfpm2* Forward | TGG TGA TGC AGA AGT TGG AG | |  |
|  | *pfpm2* Reverse | TGG GAC CCA TAA ATT AGC AGA | |  |
|  | β-*tubulin* Forward | TGA TGT GCG CAA GTG ATC C | |  |
|  | β-*tubulin* Reverse | TCC TTT GTG GAC ATT CTT CCT C | |  |
