# Supplementary material for: Piperaquine resistant Cambodian Plasmodium falciparum clinical isolates: in vitro genotypic and phenotypic characterization
Source: Malar J. 2020 Jul 25;19:269. doi: 10.1186/s12936-020-03339-w (PMC7382038; doi:10.1186/s12936-020-03339-w)
Supplement: Supplementary file 3 — Additional file 3: Table S3. Mean ± S.D of % Survival rate of Ring-stage Survival Assay (RSA) and Piperaquine Survival Assay (PSA). [file 12936_2020_3339_MOESM3_ESM.docx]

**Table S3.**

| **Sample** | **% RSA survival rate** | **% PSA survival rate** |
| --- | --- | --- |
| **W2** | 0.22 ± 0.09 | 0.46 ± 0.1 |
| **IPC-4884** | 3.89 ± 0.09 | 0 ± 0 |
| **IPC-5202** | 39.34 ± 9.6 | 0 ± 0 |
| **1** | 8.09 ± 3.8 | 59.92 ± 5.6 |
| **2** | 18.15 ± 8.1 | 43.76 ± 16 |
| **3** | 12.79 ± 1.6 | 51.34 ± 2.0 |
| **4** | 7.82 ± 0.4 | 26.38 ± 2.1 |
| **5** | 11.23 ± 3.0 | 41.59 ± 14 |
| **6** | 9.17 ± 1.9 | 40.47 ± 6.4 |
| **7** | 19.44 ± 0.9 | 29.81 ± 0.1 |
| **8** | 14.31 ± 4.9 | 45.50 ± 14 |
| **9** | 6.26 ± 1.3 | 48.51 ± 2.4 |
| **10** | 13.54 ± 1.9 | 35.15 ± 0.1 |
| **11** | 41.94 ± 11.8 | 34.25 ± 0.1 |
| **12** | 21.06 ± 5.6 | 42.80 ± 15 |
| **13** | 4.94 ± 0.9 | 0.37 ± 0.5 |
| **14** | 0 ± 0 | 0.12 ± 0.1 |
| **15** | 7.28 ± 0.6 | 22.5 ± 5.1 |
| **16** | 0.34 ± 0.3 | 28.6 ± 9.2 |
| **17** | 5.82 ± 1.7 | 0.21 ± 0.1 |
