# Supplementary material for: Piperaquine resistant Cambodian Plasmodium falciparum clinical isolates: in vitro genotypic and phenotypic characterization
Source: Malar J. 2020 Jul 25;19:269. doi: 10.1186/s12936-020-03339-w (PMC7382038; doi:10.1186/s12936-020-03339-w)
Supplement: Supplementary file 4 — Additional file 4: Table S4. IC50s of asexual stage P. falciparum parasites for different antimalarial drugs (mean ± S.D). N/A is not-applicable. [file 12936_2020_3339_MOESM4_ESM.docx]

**Table S4.**

| **ID** | **IC_50_ (nM)** | | | | | | | | | **IC_90_ (nM)** |
| --- | --- | --- | --- | --- | --- | --- | --- | --- | --- | --- |
|  | **AS** | **DHA** | **DOX** | **ATQ** | **CYC** | **LUM** | **QN** | **CQ** | **MQ** | **PPQ** |
| **W2** | 5.82 ± 1.7 | 3.06 ± 0.7 | 5308 ± 1798 | 2.19 ± 0.9 | 3334 ± 346 | 2.32 ± 0.6 | 211 ± 25 | 317 ± 75 | 51 ± 12 | 88 ± 27 |
| **D6** | 5.97 ± 1.7 | 2.63 ± 0.2 | 8149 ± 1213 | 0.67 ± 0.4 | 3.2 ± 0.8 | 10 ± 2 | 58 ± 23 | 23 ± 5 | 93 ± 21 | 51 ± 9 |
| **C2B** | 3.31 ± 0.2 | 2.42 ± 0.7 | 8290 ± 5390 | 5127 ± 485 | 29985 ± 6195 | 5.72 ± 3.3 | 178 ± 90 | 211 ± 66 | 112 ± 35 | 94 ± 43 |
| **1** | 4.82 ± 2.1 | 4.28 ± 0.8 | 11814 ± 951 | 1.15 ± 0.6 | 26445 ± 9674 | 2.30 ± 0.1 | 204 ± 30 | 445 ± 20 | 65 ± 3 | N/A |
| **2** | 4.00 ± 0.1 | 4.32 ± 0.2 | 14245 ± 3186 | 2.38 ± 0.4 | 11175 ± 909 | 2.58 ± 0.7 | 120 ± 13 | 109 ± 11 | 89 ± 6 | N/A |
| **3** | 3.22 ± 0.3 | 2.19 ± 0.9 | 11175 ± 1795 | 0.56 ± 0.2 | 9073 ± 2735 | 6.30 ± 4.4 | 82 ± 29 | 88 ± 17 | 87 ± 51 | 80918 ± 0.0 |
| **4** | 5.85 ± 1.6 | 3.79 ± 1.6 | 24105 ± 2400 | 2.24 ± 0.0 | 30585 ± 33028 | 4.35 ± 1.5 | 122 ± 139 | 451 ± 0.0 | 42 ± 0.0 | 75673 ± 0.0 |
| **5** | 3.30 ± 1.1 | 3.86 ± 1.9 | 14441 ± 922 | 0.96 ± 0.6 | 8008 ± 1169 | 0.85 ± 0.2 | 77 ± 5 | 192 ± 59 | 18 ± 3 | N/A |
| **6** | 4.35 ± 0.8 | 4.01 ± 0.8 | 25668 ± 4756 | 1.77 ± 1.1 | 15932 ± 15091 | 2.54 ± 0.8 | 125 ± 67 | 223 ± 112 | 23 ± 24 | N/A |
| **7** | 5.69 ± 0.6 | 3.89 ± 0.5 | 20899 ± 1874 | 1.22 ± 0.2 | 13559 ± 465 | 13 ± 5 | 524 ± 53 | 435 ± 36 | 173 ± 43 | 392 ± 56 |
| **8** | 2.31 ± 0.1 | 1.24 ± 0.02 | 9922 ± 1346 | 0.66 ± 0.3 | 8530 ± 784 | 1.92 ± 0.2 | 71 ± 6 | 145 ± 3 | 28 ± 10 | 94321 ± 0.0 |
| **9** | 5.31 ± 1.3 | 3.34 ± 0.3 | 24067 ± 1289 | 1.84 ± 0.7 | 12031 ± 7195 | 5.52 ± 2.3 | 133 ± 60 | 104 ± 20 | 64 ± 35 | 68439 ± 35840 |
| **10** | 4.11 ± 0.6 | 4.84 ± 0.3 | 10919 ± 1031 | 2.49 ± 0.7 | 11024 ± 2429 | 2.38 ± 0.3 | 101 ± 14 | 112 ± 32 | 70 ± 14 | 90397 ± 0 |
| **11** | 3.63 ± 0.8 | 3.24 ± 0.9 | 12815 ± 2756 | 1.22 ± 0.0 | 19067 ± 11258 | 1.87 ± 0.1 | 128 ± 20 | 295 ± 74 | 36 ± 13 | 35129 ± 6305 |
| **12** | 3.77 ± 0.7 | 2.95 ± 1.1 | 16108 ± 5158 | 1.27 ± 0.2 | 5470 ± 603 | 3.07 ± 1.4 | 159 ± 81 | 176 ± 52 | 28 ± 21 | 42330 ± 25605 |
| **13** | 6.21 ± 1.5 | 6.17 ± 1.2 | 18830 ± 2768 | 2.15 ± 0.5 | 1435 ± 822 | 29 ± 6.8 | 585 ± 63 | 859 ± 123 | 176 ± 53 | 185 ± 12 |
| **14** | 4.93 ± 0.1 | 3.09 ± 0.6 | 21113 ± 1295 | 3.23 ± 0.2 | 716.6 ± 25 | 32 ± 6.9 | 654 ± 23 | 479 ± 4 | 281 ± 15 | 172 ± 9 |
| **15** | 7.07 ± 1.6 | 7.50 ± 0.4 | 19342 ± 2555 | 3.09 ± 0.5 | 8784 ± 316 | 6.49 ± 3.7 | 172 ± 0.0 | 266 ± 0.0 | N/A | N/A |
| **16** | 4.07 ± 0.6 | 3.56 ± 0.5 | 12825 ± 850 | 2.07 ± 0.5 | 13403 ± 12586 | 1.34 ± 0.6 | 90 ± 28 | 195 ± 99 | 6.2 ± 4 | 32657 ± 45971 |
| **17** | 4.22 ± 0.2 | 3.51 ± 0.3 | 17127 ± 1247 | 0.49 ± 0.01 | 1011 ± 125 | 23 ± 3 | 356 ± 54 | 717 ± 151 | 196 ± 3 | 81 ± 0 |
