# Supplementary figures and images for: Piperaquine resistant Cambodian Plasmodium falciparum clinical isolates: in vitro genotypic and phenotypic characterization
Source: Malar J. 2020 Jul 25;19:269. doi: 10.1186/s12936-020-03339-w (PMC7382038; doi:10.1186/s12936-020-03339-w)

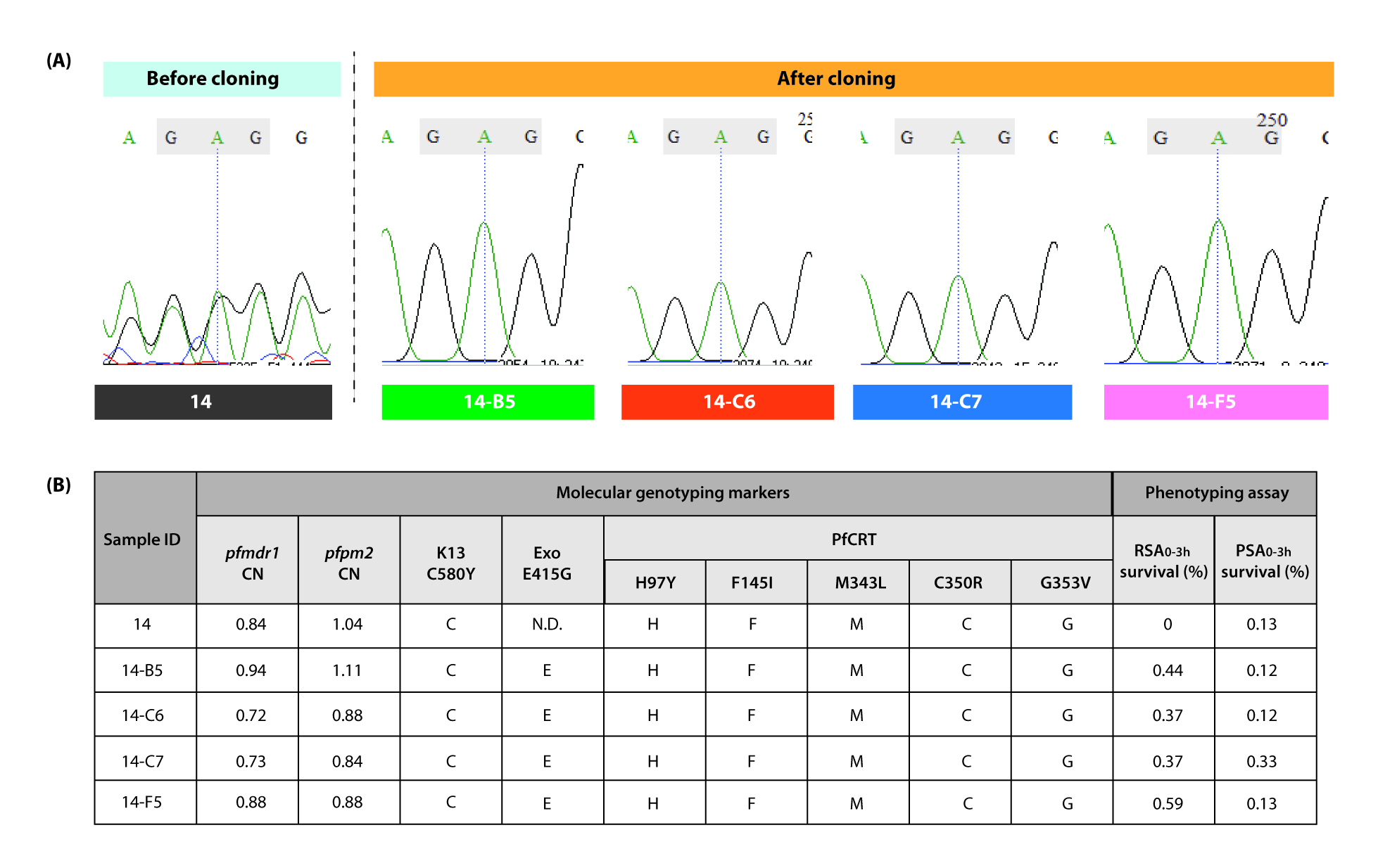

Supplement: Supplementary file 5 — Additional file 5: Figure S1. Characterization of a new field clone of P. falciparum isolate. (A) Sequencing chromatogram of pfexo gene confirmed the amino acid at position 145 of Exonuclease from P. falciparum Cambodian isolate 14 is glutamic acid (E), which is coded by a codon GAG. (B) Table represents molecular genotyping of P. falciparum Cambodian isolate 14 before and after cloning. The copy number of pfmdr1 and pfpm2 less than 1.5 and 1.6 indicates a single copy of the gene, respectively, while % RSA and % PSA cut off less than 1 and 10 represents the ART- and PPQ-sensitive, respectively. N.D stands for not determined. [file 12936_2020_3339_MOESM5_ESM.tif]
