# Supplementary material for: Piperaquine resistant Cambodian Plasmodium falciparum clinical isolates: in vitro genotypic and phenotypic characterization
Source: Malar J. 2020 Jul 25;19:269. doi: 10.1186/s12936-020-03339-w (PMC7382038; doi:10.1186/s12936-020-03339-w)
Supplement: Supplementary file 6 — Additional file 6: Table S5. Comparison of the in vitro susceptibility of Plasmodium falciparum Cambodian isolate 14 before and after cloning as well as P. falciparum 3D7 and W2 exposed to established antimalarial agents. IC50 values report the mean ± S.D from at least 3 experiments. Statistically significant difference relative to 3D7 is indicated with one asterisk (0.05 > p > 0.01). N/A is non-applicable. [file 12936_2020_3339_MOESM6_ESM.docx]

**Table S5.**

| **Compound** | **IC_50_ values (nM)** | | | | | | | **p-value** |
| --- | --- | --- | --- | --- | --- | --- | --- | --- |
|  | **3D7** | **W2** | **14** | **14-B5** | **14-C6** | **14-C7** | **14-F5** |  |
| **Dihydroartemisinin** | 7.71 + 1.16 | 3.05 + 0.39* | 3.10 + 0.33* | 4.75 + 0.25 | 4.07 + 0.11 | 4.04 + 0.83 | 5.21 + 0.11 | 0.011 |
| **Artesunate** | 5.74 + 2.16 | 4.48 + 0.58 | 3.64 + 0.06 | 2.79 + 0.12 | 3.75 + 0.30 | 3.77 + 0.61 | 3.39 + 0.41 | 0.252 |
| **Mefloquine** | 105 + 17.8 | 54.2 + 9.99 | 192 + 5.94 | 163 + 35.4 | 161.6 + 27.5 | 146 + 18.0 | 95.8 + 6.20 | 0.020 |
| **Quinine** | 72.1 + 0.00 | 218 + 12.5 | 238 + 4.78 | 283 + 77.9 | 418 + 21.4* | 541 + 26.4* | 386 + 17.0 | 0.007 |
| **Chloroquine** | 21.4 + 2.83 | 184 + 11.4 | 264 + 1.15 | 321 + 88.0 | 520 + 29.0* | 523 + 79.9* | 430 + 35.3 | 0.012 |
| **Lumefantrine** | 9.36 + 1.91 | 4.52 + 2.52 | 17.2 + 2.16 | 7.38 + 3.10 | 11.2 + 1.08 | 10.9 + 3.98 | 11.0 + 0.79 | 0.140 |
| **Piperaquine** | 81.8 + 14.3 | 67.3 + 4.39 | 82.9 + 4.48 | 71.1 + 19.8 | 80.4 + 11.2 | 79.8 + 9.02 | 86.4 + 6.19 | 0.712 |
| **Atovaquone** | 574 + 566 | 18.4 + 8.05 | 2.50 + 0.08 | 4.29 + 1.70 | 6.40 + 1.05 | 12.1 + 2.45 | 7.77 + 0.87 | 0.066 |
| **Doxycycline** | 7987 + 1942 | 6392 + 2503 | 12484 + 441 | 11564 + 1790 | 10565 + 1439 | 11507 + 3024 | 11980 + 555 | 0.291 |
| **Cycloguanil** | N/A | 2637 + 527 | 707 + 14.5 | 725 + 127 | 1024 + 38.0 | 1409 + 166 | 1405 + 481 | 0.036 |
