# Supplementary material for: Piperaquine resistant Cambodian Plasmodium falciparum clinical isolates: in vitro genotypic and phenotypic characterization
Source: Malar J. 2020 Jul 25;19:269. doi: 10.1186/s12936-020-03339-w (PMC7382038; doi:10.1186/s12936-020-03339-w)
Supplement: Supplementary file 7 — Additional file 7: Table S6. In vitro drug combination assay for 9 combinations in asexual stages of different P. falciparum strains. DHA, dihydroartemisinin; CQ, chloroquine; MQ, mefloquine; PPQ, piperaquine; ATQ, atovaquone; PG, proguanil; TQ, tafenoquine; PND, pyronaridine. ΣFIC50 (50% Fractional Inhibitory Concentrations), synergism when ΣFIC50 ≤ 0.5; toward synergism when ΣFIC50 < 1; additive when ΣFIC50 = 1; toward antagonism when ΣFIC50 > 1; antagonism when ΣFIC50 ≥ 2 to 4. The values show the mean ± S.D of 3 independent assays for each parasite line. [file 12936_2020_3339_MOESM7_ESM.docx]

**Table S6.**

| **Drug Combination** | **Mean ΣFIC_50_ of *P. falciparum* strain** | | | | | |
| --- | --- | --- | --- | --- | --- | --- |
|  | **3D7** | **W2** | **D6** | **C2B** | **IPC-5202** | **14-B5** |
| **DHA-PPQ** | 4.96 ± 1.88 | 2.05 ± 0.10 | 2.46 ± 0.37 | 1.95 ± 0.13 | 2.45 ± 0.44 | 2.15 + 0.22 |
| **CQ-CQ** | 1.08 ± 0.09 | 1.00 ± 0.02 | 1.08 ± 0.07 | 1.04 ± 0.13 | 1.09 ± 0.01 | 1.02 + 0.09 |
| **ATQ-PG** | 0.23 ± 0.06 | 0.31 ± 0.05 | 0.24 ± 0.02 | 0.16 ± 0.06 | 0.08 ± 0.05 | 0.26 + 0.11 |
| **PND-ATQ** | 3.89 ± 0.28 | 2.32 ± 0.87 | 2.87 ± 0.74 | 1.69 ± 0.26 | 3.37 ± 1.43 | 2.77 + 0.58 |
| **MQ-ATQ** | 1.43 ± 0.26 | 2.06 ± 0.68 | 1.23 ± 0.23 | 0.65 ± 0.22 | 1.34 ± 0.18 | 3.21 + 0.67 |
| **TQ-ATQ** | 0.91 ± 0.34 | 1.86 ± 0.55 | 0.69 ± 0.19 | 0.89 ± 0.13 | 1.17± 0.20 | 2.07 + 0.01 |
| **PND-PG** | 1.26 ± 0.10 | 0.95 ± 0.04 | 1.00 ± 0.10 | 0.69 ± 0.18 | 0.75 ± 0.26 | 3.42 + 0.73 |
| **MQ-PG** | 0.90 ± 0.02 | 0.90 ± 0.09 | 0.89 ± 0.08 | 0.72 ± 0.07 | 0.82 ± 0.01 | 0.84 + 0.05 |
| **TQ-PG** | 0.49 ± 0.13 | 1.86 ± 0.58 | 0.63± 0.07 | 0.82 ± 0.01 | 1.03 ± 0.08 | 2.41 + 0.45 |
